# Identifying key inflammatory genes in psoriasis via weighted gene co-expression network analysis: Potential targets for therapy

**DOI:** 10.17305/bb.2024.10327

**Published:** 2024-10-01

**Authors:** Huidan Li, Xiaorui Wang, Jing Zhu, Bingzhe Yang, Jiatao Lou

**Affiliations:** 1 Clinical Laboratory Medicine Center, Shanghai General Hospital, Shanghai, China; 2 Clinical Laboratory Medicine Center, Jiading Branch of Shanghai General Hospital, Shanghai, China; 3 School of Medicine, Shanghai Jiao Tong University, Shanghai, China

**Keywords:** Psoriasis, hub gene, inflammation-related genes, chemokine, immunohistochemistry, weighted gene co-expression network analysis (WGCNA).

## Abstract

Psoriasis is a globally prevalent chronic inflammatory skin disease. This study aimed to scrutinize the hub genes related to inflammation and potential molecular mechanisms in psoriasis. Utilizing mRNA expression profiles from public datasets GSE13355, GSE78097, and GSE14905, we set up a comprehensive analysis. Initially, we selected differentially expressed genes (DEGs) from psoriasis and control samples in GSE13355, followed by calculating inflammatory indices using genomic set variation analysis (GSVA). Weighted gene co-expression network analysis (WGCNA) was then applied to link significant modules with the inflammatory index. This process helped us identify differentially expressed inflammation-related genes (DE-IRGs). A protein–protein interaction (PPI) network was established, with the molecular complex detection (MCODE) plug-in pinpointing six chemokine genes (*CCR7*, *CCL2*, *CCL19*, *CXCL8*, *CXCL1*, and *CXCL2*) as central hub genes. These genes demonstrated pronounced immunohistochemical staining in psoriatic tissues compared to normal skin. Notably, the *CCR7* gene exhibited the highest potential for m6A modification sites. Furthermore, we constructed transcription factor (TF)-microRNA-mRNA networks, identifying 139 microRNAs and 52 TFs associated with the hub genes. For the LASSO logistic regression model, the area under the curve (AUC) in the training set was 1, and in the two validation cohorts GSE78097 and GSE14905, it was 1 and 0.872, respectively. In conclusion, our study highlights six chemokine genes (*CCR7*, *CCL2*, *CCL19*, *CXCL8*, *CXCL1*, and *CXCL2*) as potential biomarkers in psoriasis, providing insights into the immune and inflammatory responses as pivotal instances in disease pathogenesis. These findings pave the way for exploring new therapeutic targets, particularly focusing on chemokine-associated pathways in psoriasis treatment.

## Introduction

Psoriasis, a chronic immune-mediated inflammatory disease, is characterized by distinct skin lesions and affects an estimated 120 million children and adults worldwide [1, 2]. This condition extends beyond dermatological issues, encompassing systemic problems, and is often associated with comorbidities, such as psoriatic arthritis, metabolic syndrome, depression, and cardiovascular diseases [3]. Plaque psoriasis, which accounts for over 90% of cases is the most prevalent clinical phenotype [1]. However, the broad clinical manifestations of psoriasis often lack specific biomarkers, posing challenges for accurate diagnosis and effective treatment [4]. Current therapeutic strategies, primarily aimed at symptom management, offer limited long-term effectiveness and are associated with significant side effects. Conventional treatments, such as corticosteroids and vitamin D analogs, are recommended only for mild psoriasis cases and carry risks with prolonged use [5]. Although biological agents have been proven beneficial for a certain patient group, they introduce potential complications related to immune system modification [6]. This scenario underscores the need for novel therapeutic approaches built on a more comprehensive understanding of psoriasis pathophysiology.

The pathogenesis of psoriasis is complex, involving both the innate and adaptive immune systems. Central to this process are dendritic cells (DCs), macrophages, neutrophils, NK cells, and especially T cells [7, 8]. Activated keratinocytes release cytokines and chemokines, which, along with these inflammatory cells, drive the characteristic alterations in skin and blood vessels seen in psoriasis [9, 10]. Consistently, chemokines and their receptors are pivotal in the inflammatory milieu, guiding the movement and activation of T lymphocytes, monocytes, and neutrophils [11, 12]. Although several chemokines, such as CCL13, CXCL12, CXCL10, CCL27, and CCR6, have been identified in the context of psoriasis [11, 13], CXCL10, in particular, has been suggested as a biomarker for psoriasis progression [14]. The deletion of CCR6 in mice has been shown to impede the development of a psoriasis-like phenotype following IL-23 injection [15]. Despite these insights, the precise mechanism by which chemokines facilitate psoriasis pathogenesis is not fully understood, underlining a crucial area for further research. Therefore, this study aims to explore the existing gaps regarding specific chemokine gene networks.

Recent advances in gene expression profiling have illuminated the pathogenesis of psoriasis [16, 17]. Weighted gene co-expression network analysis (WGCNA), a robust method for associating genes with phenotypic traits, played a key role in revealing gene networks pertinent to psoriasis [18–20]. By integrating WGCNA with differential expression analysis, Ahn et al. [20] uncovered various psoriasis-related networks of coding and non-coding genes, while Sundarrajan et al. [21] identified potential diagnostic genes for psoriasis. Nonetheless, WGCNA has yet to be applied in identifying networks of inflammation-related genes.

In our study, we aimed to address these gaps by using WGCNA in conjunction with an inflammation index to identify differentially expressed inflammation-related genes (DE-IRGs) in psoriasis. Subsequently, we identified potential biomarkers, focusing on inflammation-associated hub genes, and assessed their predictive sensitivity and specificity for distinguishing psoriasis from control subjects. Importantly, we pinpointed transcription factors (TFs), miRNAs, and potential drug candidates that interact with these hub genes. These findings provide significant insights into the role of inflammation genes in psoriasis and propose novel biomarkers for targeted therapeutic interventions. They enhance our understanding of psoriasis pathogenesis, provide essential guidance for personalized therapy and drug development, and, finally, markedly contribute to the advancement of psoriasis management strategies and treatment options.

## Materials and methods

### Data source

The datasets GSE13355 [22], GSE14905 [23], and GSE78097 [24] were obtained from the Gene Expression Omnibus (GEO) database (https://www.ncbi.nlm.nih.gov/gds). In this study, the GSE13355 dataset, which was derived from the chip data of the GPL570 platform, included 58 psoriasis and 64 control samples. The GSE14905 and GSE78097 datasets were also based on the GPL570 platform chip data and consisted of 28 and 27 psoriasis and 21 and 6 control samples, respectively. For this study, GSE13355 was utilized as a training set, while GSE14905 and GSE78097 were employed as external validation sets. The GSE13355 raw data from 180 microarrays were processed using the robust multichip average (RMA) method. The expression values in the table were after adjustment of RMA expression values (on the log scale) to account for batch and sex effects. GSE78097 was processed using GCRMA (gcrma package using R/Bioconductor) and adjusted for batch effects. GSE14905 analyzed data using ArrayAssist Lite. Moreover, the gene expression patterns of IRGs (HALLMARK_INFLAMMATORY_RESPONSE, Table S1) were acquired from the Molecular Signature Database (MSigDB) database (https://ngdc.cncb.ac.cn/databasecommons).

### Screening of differentially expressed genes (DEGs)

The limma package (v3.52.4) [25] was used to identify the DEGs between the psoriasis and control samples in the GSE13355 dataset. The cutoff values were adjusted *P* < 0.05 and ׀*log*_2_ *fold change* (*FC*)׀ > 1. The results were visualized on heatmap and volcano plots using ggplot2 (v3.3.6) [26] and pheatmap packages (v1.0.12) [27], respectively.

### Weighted gene co-expression network analysis (WGCNA)

Originally, the inflammatory index of each GSE13355 sample was calculated via the single-sample gene set enrichment analysis (ssGSEA) algorithm using a genomic set variation analysis (GSVA) package (v1.44.5) [28]. Then, inflammatory index-related key modulars in GSE13355 were identified via the WGCNA R package [18]. The procedure began by creating a sample clustering tree to screen for outliers, evaluating whether their removal was crucial for the credibility of the forthcoming analyses. Afterward, the selection of a soft threshold (β) for constructing the co-expression network ensued. This network construction aimed to match a scale-free R2 close to 0.85 and a mean connectivity value close to 0, thus ensuring a closer correspondence to a scale-free topology. Next, according to their proximity, genes were compared for similarity, and a phylogenetic tree of those genes was created. The dynamic tree-cutting algorithm was employed to divide the modules. Modules with significant correlation were chosen as key modules related to the inflammatory index (׀ *r* ׀ > 4, *P* < 0.05). Genes contained in these key modules were designated as key module genes related to the inflammatory index. Finally, the intersections of DEGs and index-related key module genes were obtained using the VennDiagram package (v1.7.3) [29] and termed as DE-IRGs for future analysis.

### Functional enrichment analysis

In an effort to elucidate potential biological functions and signaling pathways associated with DE-IRGs, the clusterProfiler package (v4.7.1.3) [30] handled Gene ontology (GO) and Kyoto encyclopedia of genes and genomes (KEGG) enrichment analysis (*P* < 0.05). The GO system comprises biological processes, molecular functions, and cellular components.

### PPI network construction

The protein–protein interaction (PPI) network, constructed with the assistance of the Search tool for the retrieval of interacting genes (STRING) database (http://string-db.org) [25], set the interaction with a combined score ≥ 0.4 as the cut-off point [31]. The molecular complex detection (MCODE) plug-in in Cytoscape software aided in the identification of hub modules with default parameters (cutoff degree ═ 2, node score cutoff ═ 0.2, k-core ═ 2, and max depth ═ 100). Hub module genes were defined as hub genes in this study, with their correlations analyzed by the Corrplot package (v0.92) [32]. Lastly, the expression levels of hub genes were compared between psoriasis and control samples in the GSE13355 dataset.

### Immunohistochemical analysis

Four patients diagnosed with psoriasis vulgaris were recruited in this study. Dermatologists confirmed the diagnosis, with exclusion criteria being previous treatment, and complications, such as ulceration, bleeding, and local infection. Healthy control tissue was derived from age-matched patients who underwent plastic surgery, whose tissues would be otherwise discarded. The collected tissue samples were then fixed in a 4% paraformaldehyde solution and preserved in paraffin. The immunohistochemical (IHC) analysis included the primary antibodies against CXCL1 (12335-1-AP, Proteintech, 1:100), CXCL2 (bs-1162R, BIOSS, 1:200), CXCL8 (27095-1-AP, Proteintech, 1:200), CCL19 (13397-1-AP, Proteintech, 1:200), CCL2 (66272-1-Ig, Proteintech, 1:200), and CCR7 (GB11502, Servicebio, 1:50). Upon staining, the samples were examined microscopically, and representative parts were photographed.

### Correlation analysis between infiltrating immune cells and hub genes

The infiltration levels of 28 immune cells and immune functions in both psoriasis and control samples of the GSE13355 dataset were calculated using the ssGSEA algorithm in the GSVA package, based on the expression of 28 immune-infiltrating cell-marker genes (Table S2) [33]. In parallel, the correlation between the hub gene and 28 infiltrating immune cell subpopulations and immune function was assessed.

### Gene set enrichment analysis (GSEA) and small-molecule drug prediction

The clusterprofiler package was used to perform GSEA on hub genes, using “C2.cp.kegg.v7.0.symbols.gmt” as the reference gene set (*P* < 0.05) The SRAMP dataset (https://www.cuilab.cn/sramp) was used to predict the specific m6A site of the mRNA sequence of hub genes.

Besides that, to identify potential therapeutic drugs for psoriasis, hub genes were inputted into the Drug Gene Interaction Database (DGIdb) (https://www.dgidb.org/) [34]. The results were then visualized using Cytoscape. Afterward, the 3D protein conformations of hub genes were explored in the PDB database (https://www.rcsb.org). The active ingredient conformations were generated and downloaded through the PubChem Compound platform. CB-Dock was employed to construct docking grid boxes between hub genes and their active ingredients, while the visualization was provided by PyMOL. Lastly, the Comparative Toxicogenomics Database (CTD) (https://ctdbase.org/) was used to assess the interactions between hub genes and other diseases.

### Construction of hub genes-miRNA regulatory network

This study utilized the miRNet database (https://www.mirnet.ca/) [35] to identify miRNAs and TFs associated with hub genes. Subsequently, the miRNA-TF-hub gene network was constructed using Cytoscape. Additionally, CTD was employed to investigate interactions between hub genes and other diseases.

### Receiver operating characteristic (ROC) curve analysis

To assess the significance of hub genes in psoriasis and their ability to differentiate psoriasis patients from the control group in the GSE13355 dataset, we performed ROC curve analysis and calculated the area under the curve (AUC) using the “pROC” package [36]. A hub gene was considered capable of distinguishing psoriasis from the control group with high specificity and sensitivity when the AUC value exceeded 0.7 [37]. The ROC curve employed a resampling technique (Bootstrap) to test the accuracy of the predictive model, which is an important statistical method in nonparametric statistics used for estimating the variance of a statistic and thus for interval estimation.

### Establishment of the least absolute shrinkage and selection operator (LASSO) model

We applied the LASSO algorithm using the “glmne” package (v4.1.7) [38] and screened the gene signatures under the optimal λ value with the smallest classification error. By employing ten-fold cross-validation, we effectively assessed the model’s performance, enabling the selection of optimal regularization parameters. This process enhanced the model’s generalization capability and stability. The performance of the LASSO-based model in the GSE13355 dataset was evaluated using the ROC curves, decision curve analysis (DCA), and precision–recall (P–R) curves. At the same time, GSE14905 and GSE78097 were set as the validation cohorts for the discriminative performance of the gene signatures.

### Ethical statement

The study was conducted in accordance with the Declaration of Helsinki and approved by the Ethics Committee of Shanghai General Hospital (Protocol No: 2022SQ099 as of February 28, 2022). All patients provided written informed consent.

## Results

### Screening of DEGs

**Figure 1.**
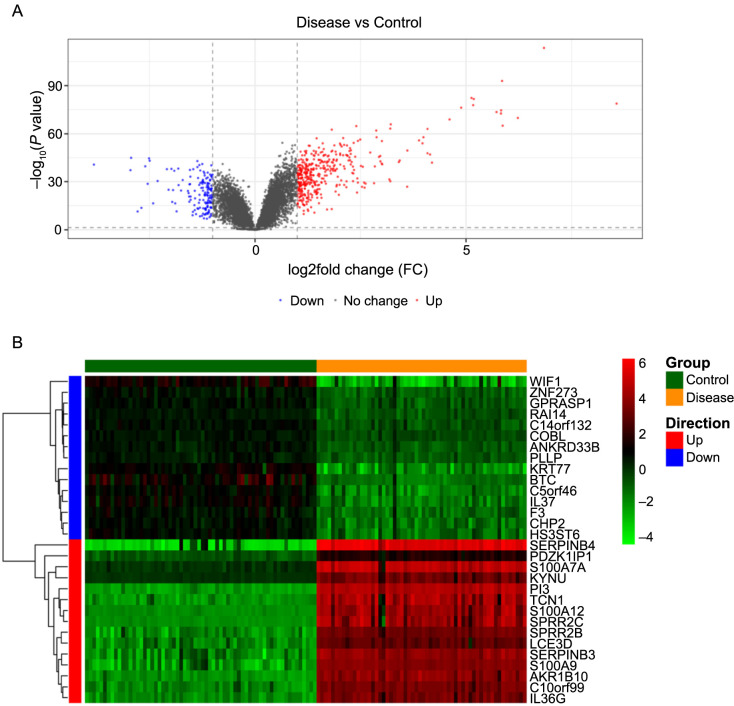
**The volcano plot and heatmap of the aberrantly expressed mRNAs in the GSE13355.** (A) Volcano plot of GSE13355; (B) Heatmap of GSE13355. The adjusted *P* value (false discovery rate) < 0.05 and log_2_-FC > 1 were used as the cutoff criteria to screen DEGs. The 15 most significantly upregulated genes and the most significantly downregulated genes were selected for heat map visualization. log_2_-FC: log_2_-fold change; DEGs: Differentially expressed genes.

In our analysis of the GSE13355 database, we thoroughly analyzed the gene expression of both psoriasis and control samples. We identified a total of 561 DEGs, comprising 405 upregulated and 156 downregulated genes (when comparing psoriasis vs control conditions) (Figure 1A). A heatmap depicting these DEGs is presented in Figure 1B.

### Construction of co-expression networks

**Figure 2.**
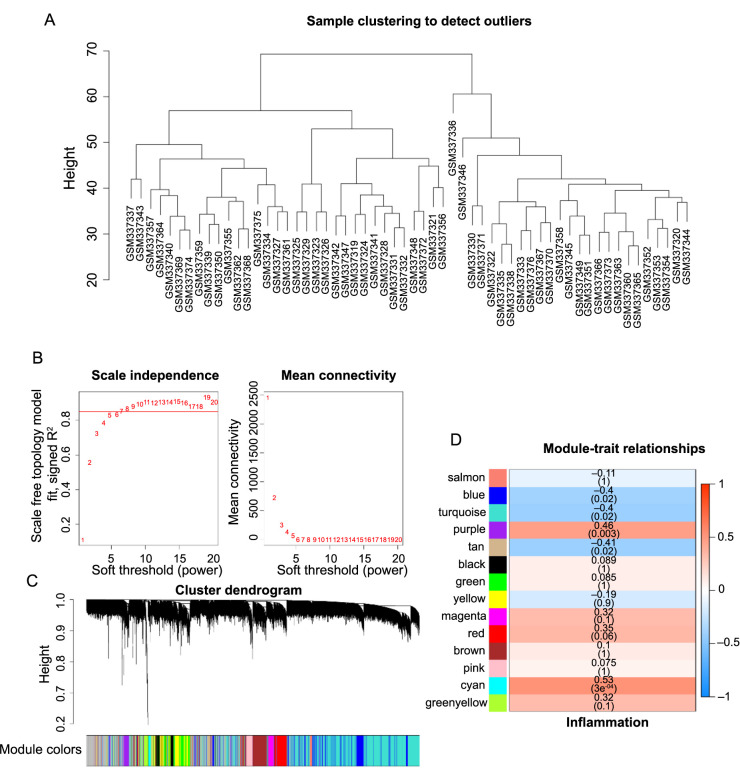
**Gene modules identified by WGCNA.** (A) Sample clustering to detect outliers; (B) Network topology for different soft-thresholding powers, β ═ 7 was selected as the best soft threshold for subsequent analysis; (C) The cluster dendrogram of genes; (D) Module–trait relationships. WGCNA: Weighted gene co expression network analysis.

The inflammatory index of the 58 psoriasis patients from the GSE13355 database was computed using the GSVA algorithm. The analysis showed the absence of an outlier sample within the GSE13355 database (Figure 2A). Consequently, the optimal soft-thresholding power was set at 7 (Figure 2B), resulting in 14 identified modules from the co-expression network (Figure 2C). Five modules (cyan, purple, blue, turquoise, and tan) significantly associated with the inflammatory index were selected for further examinations (Figure 2D).

### Functional enrichment analysis

**Figure 3.**
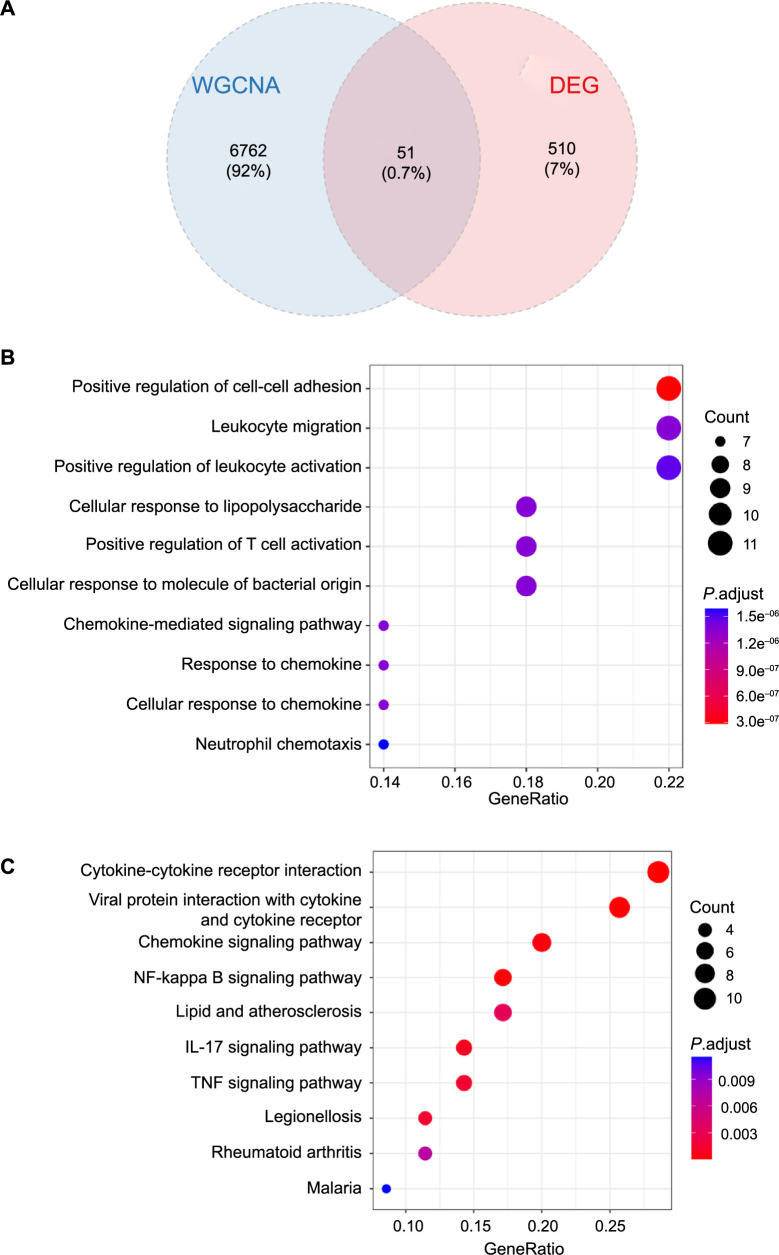
**Functional enrichment analysis of DE-IRGs.** (A) Venn diagram shows 51 DE-IRGs were identified by the intersection of DEGs and genes in key modules; (B) GO analysis of the intersecting genes; (C) Results of KEGG pathway analysis of the intersecting genes. DEG: Differentially expressed genes; DE-IRGs: Differential expressed inflammation-related genes; GO: Gene ontology; KEGG: Kyoto encyclopedia of genes and genomes; IL: Interleukin; TNF: Tumor necrosis factor; WGCNA: Weighted correlation network analysis.

A total of 51 DE-IRGs were extracted, as illustrated in the Venn diagrams (Figure 3A). The GO analysis revealed that DE-IRGs were primarily increased in the positive regulation of cell–cell adhesion, leukocyte migration, leukocyte activation, and the cellular response to lipopolysaccharide (Figure 3B). The KEGG enrichment analysis demonstrated that these DE-IRGs significantly participated in cytokine–cytokine receptor interaction, the interference of a viral protein with a cytokine and a cytokine receptor, and the chemokine signaling pathway (Figure 3C).

### PPI network and hub gene identification

**Figure 4.**
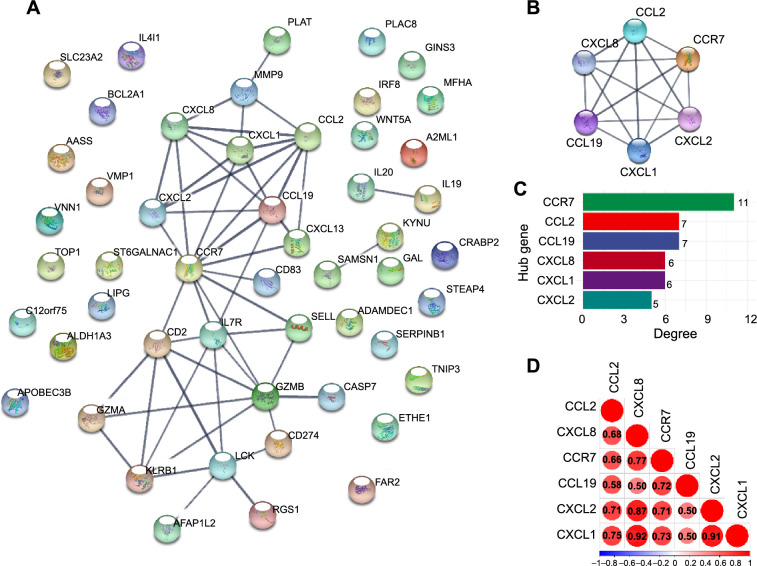
**PPI network and hub genes.** (A) The PPI network between 51 DE-IRGs was constructed. Each node represents a protein, while each edge represents one protein–protein association; (B) Interaction network among hub genes. The interaction evidence degree between proteins is presented as the gray scale of the lines; (C) Connectivity distribution between hub genes; (D) Correlation heatmap between the six hub genes. PPI: Protein–protein interaction; DE-IRGs: Differential expressed inflammation-related genes.

Six hub genes (*CCR7*, *CCL2*, *CCL19*, *CXCL8*, *CXCL1*, and *CXCL2*) were isolated from the PPI network of DE-IRGs and established using the STRING database (Figure 4A–4C). *CXCL1* and *CXCL8* showed a strong positive correlation (*r* ═ 0.92) (Figure 4D).

### Validation of hub gene expression via immunohistochemical staining analysis

**Figure 5.**
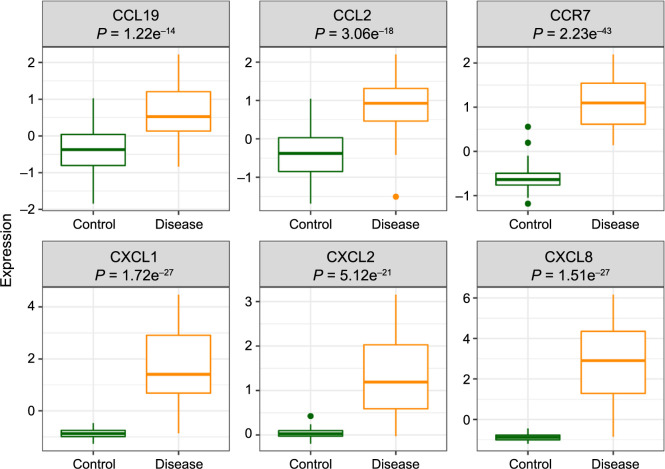
Gene expression levels of hub genes analyzed from the GSE13355 database.

**Figure 6.**
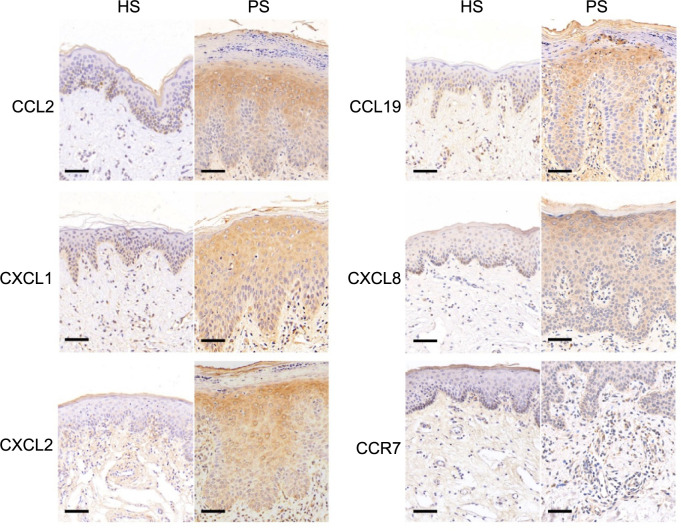
**Immunohistochemical validation of CCR7, CCL2, CCL19, CXCL8, CXCL1, and CXCL2 expression in human skin tissue.** Bar ═ 50 µm. HS: Healthy sample; PS: Psoriasis.

Genes from the GSE13355 database exhibited increased expression levels in psoriasis patients compared to healthy controls (Figure 5). To experimentally validate the reliability and accuracy of our findings, we examined the expression of hub genes through immunohistochemistry (IHC) staining. Our analysis revealed elevated expression levels of CCR7, CCL2, CCL19, CXCL8, CXCL1, and CXCL2 in psoriatic tissue compared to their levels in comparable healthy human tissue (Figure 6). Consequently, these IHC staining outcomes confirmed the enriched expression profile of hub genes in psoriatic conditions as opposed to healthy controls.

### Correlation analysis between hub genes and immune cells

**Figure 7.**
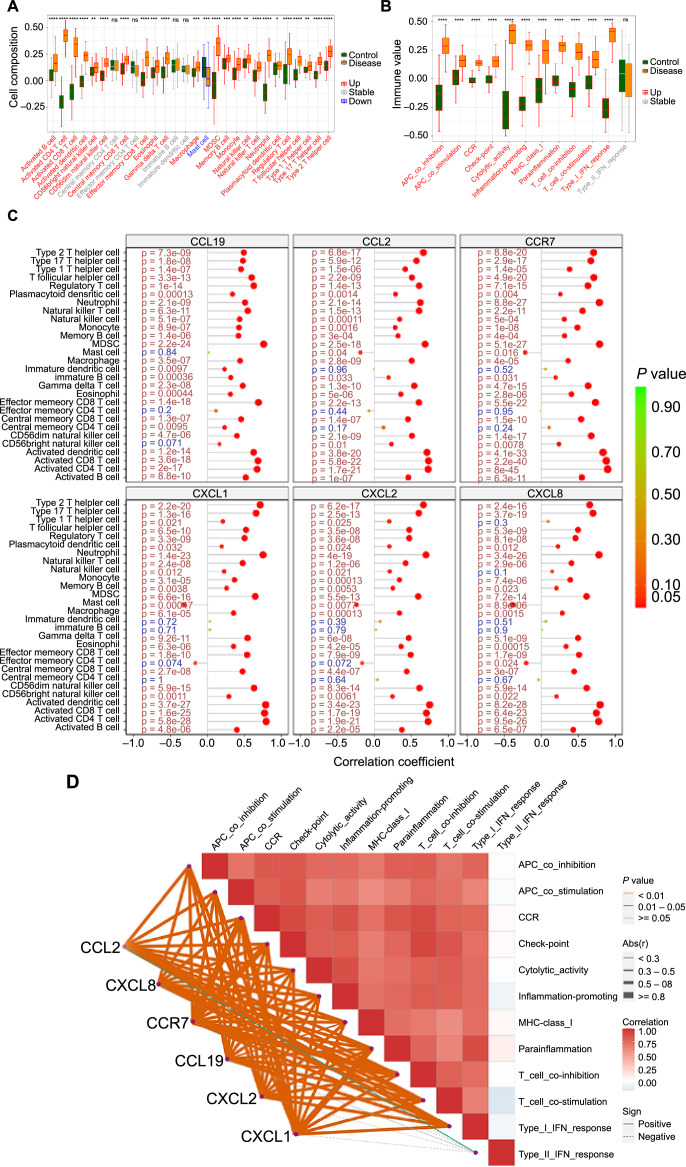
**Landscape of immune infiltration between psoriasis and normal control.** (A) The difference of immune cells and (B) immune function subtypes between psoriasis patients (yellow) and controls (green). (C) Correlation analysis of the hub genes and infiltrating immune cells and (D) immune functions. The color of the spots represents the *P* value, while the size represents the gene number (ns ═ not significant; *P< 0.05; **P <0.01; ***P < 0.001; ****P < 0.0001).

To further explore the potential molecular mechanisms through which hub genes might influence the progression of psoriasis, we assessed the relationship between immune infiltration and these hub genes within our psoriasis dataset. Our analysis, which included 28 types of immune infiltrating cells, revealed that activated B cells, activated CD4+ and CD8+ T cells, activated DCs, CD56+ bright and dim natural killer cells, central memory CD8+ T cells, effector memory CD8+ T cells, eosinophils, γ δ-T cells, macrophages, mast cells, myeloid-derived suppressor cells (MDSCs), memory B cells, monocytes, natural killer and T cells, neutrophils, plasmacytoid DCs, regulatory T cells, T follicular helper cells, type 1, 2, and 17 T helper cells varied significantly between psoriasis and healthy control groups (Figure 7A).

Additionally, several immunologic functions – such as APC co-inhibition, APC co-stimulation, CCR, check-point, cytolytic activity, inflammation-promoting, MHC class I, parainflammation, T cell co-inhibition, T cell co-stimulation, and type I interferon response – showcased marked improvement in psoriasis patients (Figure 7B). *CCL19* gene exhibited a robust positive correlation with MDSCs (*P* ═ 2.2 × 10^−24^), while *CCL2* was strongly correlated with activated CD8+ T cells (*P* ═ 5.8 × 10^−22^). Furthermore, *CCR7* (*P* ═ 8 × 10^−45^) and *CXCL1* (*P* ═ 5.8 × 10^−28^) demonstrated strong positive correlations with activated CD4+ T cells. Finally, *CXCL2* (*P* ═ 3.4 × 10^−23^) and *CXCL8* (*P* ═ 8.2 × 10^−28^) displayed significant positive correlations with activated DCs (Figure 7C and 7D).

### Gene set enrichment analysis (GSEA)

**Figure 8.**
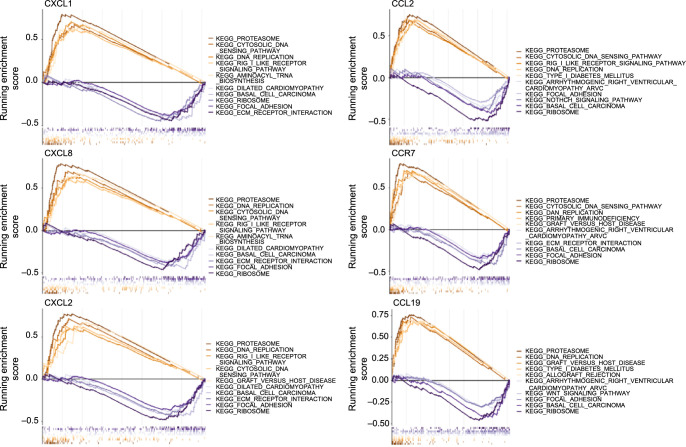
**Functional GSEA of hub genes.** GSEA: Gene set enrichment analysis; KEGG: Kyoto encyclopedia of genes and genomes.

In the subsequent analysis, the functions of our key genes were further explored through GSEA (Figure 8A–8F). In the high-expression groups of *CCR7*, *CCL2*, *CCL19*, *CXCL8*, *CXCL1*, and *CXCL2*, genes associated with proteasome and DNA replication processes were notably enriched. Conversely, the low-expression groups of these genes exhibited significant enrichment in the ribosome and focal adhesion pathways. A prediction analysis using SRAMP suggested that among the six key genes, the mRNA sequence of CCR7 held the highest potential for m6A modification sites, as supported by a very high confidence level (Figure S1).

**Figure 9.**
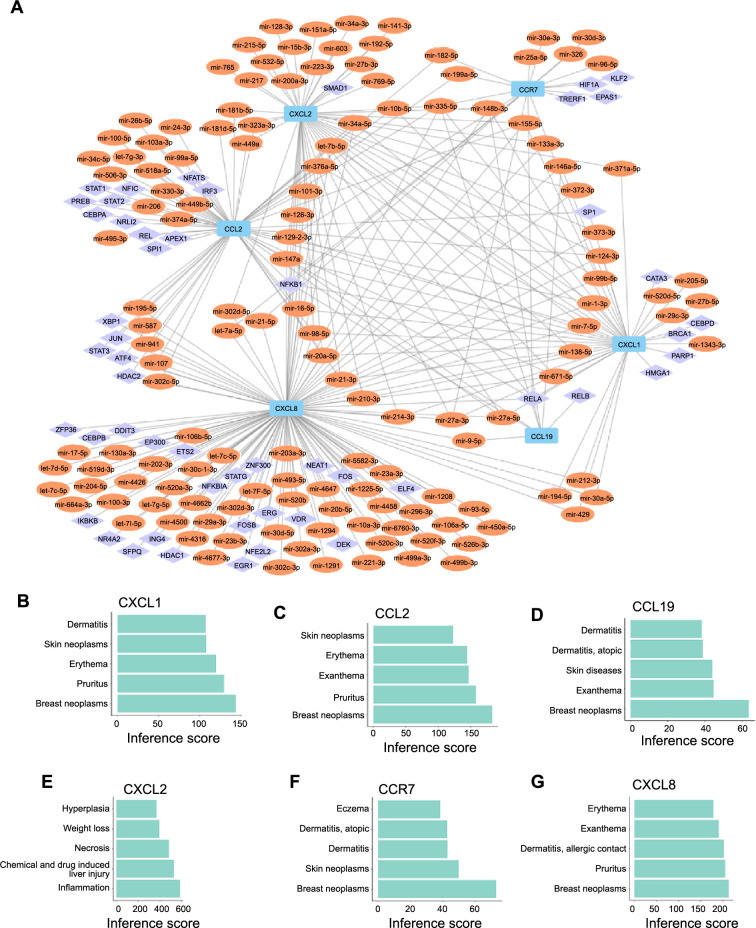
**Regulatory networks and disease prediction.** (A) Construction of the TF-miRNA-hub gene network in psoriasis based on the miRNet database; (B) The relationship between CXCL1 and other diseases; (C) The relationship between CCL2 and other diseases; (D) The relationship between CCL19 and other diseases; (E) The relationship between CXCL2 and other diseases; (F) The relationship between CCR7 and other diseases; (G) The relationship between CXCL8 and other diseases. TF: Transcription factor.

### Regulatory networks with miRNAs and gene interactions with disease

We established a TFs-miRNAs-targets regulatory network involving six hub genes, 231 miRNAs, and 86 TFs (Figure 9A). Within this network, the hub genes were denoted by blue, TFs by purple, and miRNAs by orange nodes. Remarkably, *CXCL8* emerged as the hub gene subjected to regulation by the highest number of both miRNAs (92) and TFs (39). Moreover, hsa-mir-335-5p stood out as a potentially significant miRNA, given its involvement in regulating all six key genes. Diagrams depicted in Figure 9B–9G emphasized the substantial interactions of *CCL19*, *CCL2*, *CCR7*, *CXCL1*, and *CXCL8* with breast neoplasms, while highlighting *CXCL2*’s particularly prominent interaction with inflammation.

### Screening of small molecule drugs and molecular docking

**Figure 10.**
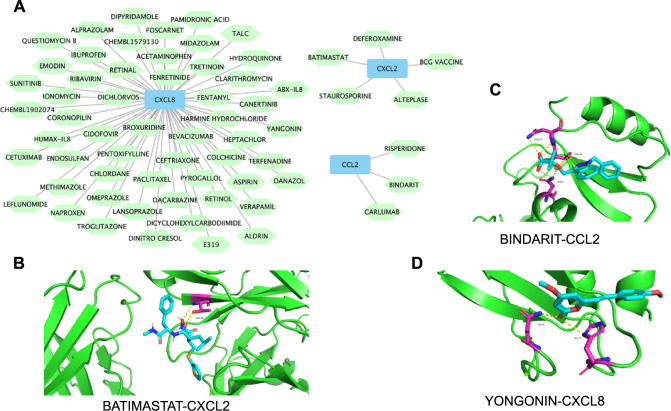
**Screening of small molecule drugs and molecular docking.** (A) Drug–gene networks. Blue squares represent hub genes; green diamonds represent drugs; (B) Molecular docking between batimastat and CXCL2; (C) Molecular docking between bindarit and CCL2; (D) Molecular docking between yangonin and CXCL8.

In this study, 64 potential therapeutic drugs for psoriasis were identified using the DGIdb database (Table S3). Notably, drugs, such as carlumab and bindarit, were discovered to target CCL2. Similarly, several drugs, including ABX-IL8, HuMax-IL8, and cetuximab, were found to interact with CXCL8. Additionally, five drugs presented potential interactions with CXCL2. However, no small molecule drugs capable of targeting CCR7, CCL19, or CXCL1 were identified in this database. This research also involved the construction of drug–gene networks by Cytoscape (Figure 10A). The resultant networks revealed that the binding energies during docking between batimastat and CXCL2 (−7.0 kcal/mol, Figure 10B), bindarit and CCL2 (−6.5 kcal/mol, Figure 10C), as well as yangonin and CXCL8 (−5.6 kcal/mol, Figure 10D), were all below −5.00 kcal/mol.

### Predictive performance of hub genes for psoriasis

**Figure 11.**
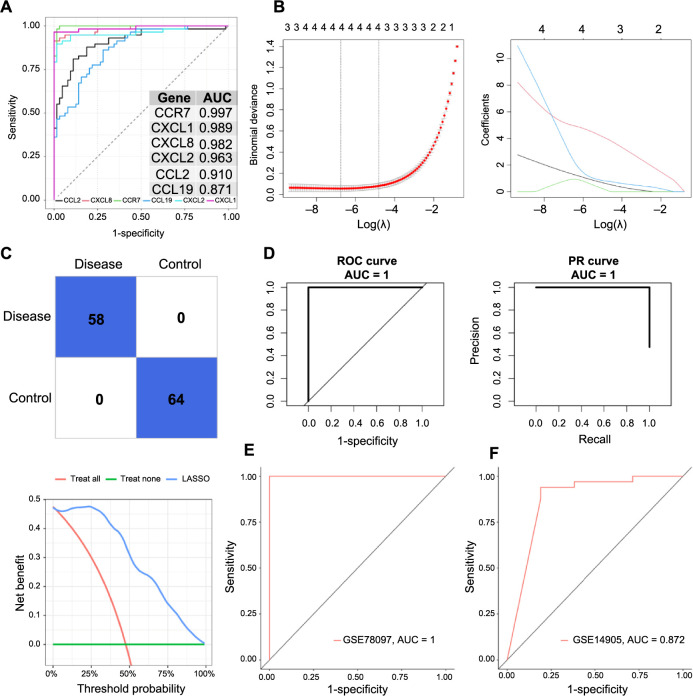
**ROC curve analysis of hub genes and gene signature.** (A) ROC curve analysis of six hub genes from GSE13355 database; (B) LASSO diagnostic model construction based on hub gene expression, including screening of the optimal parameter with λ (left) and LASSO coefficient spectrum of 4 hub genes selected by optimal (right); (C) Confusion matrix for the Lasso regression model; (D) Evaluations of the prediction model by ROC curve, P–R curve, and DCA curve; (E) The ROC curves of LASSO diagnostic model in GSE78097 and (F) GSE14905 databases. LASSO: Least absolute shrinkage and selection operator; ROC: Receiver operating curve; DCA: Decision curve analysis; P--R: Precision--recall curve.

As illustrated in Figure 11A, AUC values for *CCR7*, *CCL2*, *CCL19*, *CXCL8*, *CXCL1*, and *CXCL2* were, respectively, recorded as 0.997, 0.910, 0.871, 0.982, 0.989, and 0.963. This data strongly suggested a psoriasis-wise significant discriminatory potential of these hub genes (Figure 11A). To further refine our selection of these critical genes, we employed the LASSO algorithm (Figure 11B). This approach led to the development of the LASSO-based model that incorporated *CXCL8*, *CCR7*, *CXCL2*, and *CXCL1* (λ ═ 0.001162551). The efficacy of this model, when tested on the training set, was represented via the confusion matrix (Figure 11C). Furthermore, the ROC curve indicated the model’s high accuracy, achieving an AUC value of 1, mirroring the P–R and DCA curves (Figure 11D). Validation of the model on two external datasets (GSE78097 and GSE14905) yielded AUC values of 1 and 0.872, respectively (Figure 11E and 11F), confirming the model’s robust discriminatory power across different datasets. These findings highlight the potential of these selected hub genes as reliable biomarkers for psoriasis, offering promising directions for future diagnostic and therapeutic strategies.

## Discussion

Psoriasis is a chronic inflammatory skin disorder characterized by a complex pathogenesis that remains only partially understood [1]. Research teams worldwide have employed transcriptomic analysis to identify genes and pathways associated with the condition. For example, Ahn et al. [20] found that the majority of genes in modules significantly associated with psoriasis are lncRNAs, which are involved in lipid metabolism and olfactory receptor activity. Moreover, Choudhary et al. [39] utilized the GSE78097 dataset to identify biomarkers for mild and severe psoriasis by comparing gene expression between lesional and non-lesional skin. Additionally, GSE13355 and GSE14905 are two gene datasets extensively used for psoriasis research contributing to numerous studies [40, 41].

Building on previous efforts to identify biomarkers for psoriasis [20, 42], our study utilized WGCNA with an inflammation index as a clinical feature, making a novel approach in psoriasis research. This strategy enabled us to identify DE-IRGs as potential targets for further biomarker exploration. To elucidate the DE-IRGs’ functional importance, we conducted GO enrichment analysis, which revealed significant enrichment in biological processes such as the positive regulation of cell–cell adhesion, leukocyte migration and activation, as well as a cellular response to lipopolysaccharide. Furthermore, pathway enrichment analysis indicated that DE-IRGs were significantly involved in cytokine–cytokine receptor interaction, viral protein interaction with cytokine and cytokine receptor, and the chemokine signaling pathway. These findings not only support those reported by Li et al. [19] but also illuminate the intricate network of molecular interactions contributing to immune dysregulation in psoriasis. By building upon previous results and providing a more comprehensive analysis of DE-IRGs and their functional implications, our study contributes valuable insights to the existing body of literature, emphasizing the pivotal role of immune response and inflammation pathways in the pathogenesis of psoriasis.

Chemokines, a family of inflammatory cytokines, play a crucial role in inducing the directional migration and activation of leukocytes into inflamed tissues [12]. The chemokine and chemokine receptor system’s involvement in inflammation has become a focal point for developing therapeutic strategies for autoimmune and inflammatory diseases [43]. In our study, the PPI network of the aforementioned DE-IRGs was constructed using the STRING database, identifying six chemokine-related genes (*CCR7*, *CCL2*, *CCL19*, *CXCL8*, *CXCL1*, and *CXCL2*). Further, correlation analysis between these hub genes and 28 types of infiltrating immune cells revealed that these six potential target genes are closely and positively associated with the infiltration of MDSC, activated CD4+ T cells, activated CD8+ T cells, activated DCs, and neutrophils. These findings highlight the potential significance of the chemokine system in immune response and inflammation, suggesting its relevance as a therapeutic target in autoimmune and inflammatory diseases.

Previous research has demonstrated that CCL19 and its receptor CCR7, which are expressed by central memory/naive T cells and maturing DCs, exhibit elevated expression in the dermal aggregates of psoriasis lesions [44]. Rittié and Elder [45] have identified CCL19 and CCR7 as potential mediators of immune organization in psoriasis. The CCL19/CCR7 axis plays a crucial role in maintaining the balance between immunity and tolerance by regulating naive and regulatory T cells as well as the migration of DCs in lymphoid organs [44]. Meanwhile, CCL19 is vital for sustaining naive T cell survival in vitro, and mice lacking CCL19 exhibit reduced T cell viability in vivo [46]. This situation highlights the crucial role of CCL19 and CCR7 in the progression of psoriasis. In line with these observations, our study discovered that the expression of CCL19 and CCR7 was upregulated in patients with psoriasis, suggesting that they may serve as biomarkers for the disease (AUC ═ 0.871, AUC ═ 0.997, respectively).

CXCL1, CXCL2, and CXCL8 belong to the subfamily of neutrophil-activating chemokines, which function through the activation of CXCR1 and CXCR2 receptors. These receptors are present on various leukocytes, including neutrophils, T cells, monocytes, DCs, natural killer cells, mast cells, and MDSCs [47]. The presence of neutrophils in injured skin lesions is a notable histological feature of psoriasis [1]. Hueber et al. [48] demonstrated that IL-33 is instrumental in directing neutrophils to lesion sites, partly due to increased CXCL1 expression in a psoriasis mouse model. Furthermore, several studies have demonstrated that CXCL8 not only facilitates cell recruitment directly but also contributes to angiogenesis within the dermal microvasculature, particularly during psoriasis’ chronic phase, thus indirectly supporting cellular migration by providing nutrients and oxygen [49]. Single-cell sequencing revealed that CCR1+ macrophages prominently express genes linked to inflammation and chemotaxis, notably CXCL8 and CXCL2 [13]. Our research revealed that elevated levels of CXCL1, CXCL2, and CXCL8 in psoriasis patients are significantly associated with the infiltration of immune cells such as T cells, neutrophils, and DCs, hence providing considerable diagnostic value with an AUC of 0.989, 0.963, and 0.982, respectively.

Numerous studies have documented increased CCL2 expression in both the skin lesions and peripheral blood of individuals with psoriasis and other skin conditions [50]. CCL2, secreted by keratinocytes, recruits circulating monocytes to the inflamed skin in a CCR2-dependent manner, where they differentiate into macrophages [50]. Interestingly, Wang et al. [51] discovered that injecting CCL2 into non-lesional areas could induce psoriasis-like skin inflammation along with TNF-α, whereas TNF-α injection alone did not cause inflammation, underscoring CCL2’s crucial role in the development and persistence of psoriatic skin disease. Consistent with these findings, our study also observed an upregulation of CCL2 in psoriatic skin lesions, identifying it as a valuable inflammatory biomarker for differentiating psoriasis from controls, with an AUC of 0.910.

Recent studies have increased awareness of MDSCs in the pathogenesis of psoriasis [52]. MDSCs, characterized as a heterogeneous group of immature myeloid cells with immunoregulatory functions, have been noted to accumulate in both the peripheral blood and lesional skin of psoriasis patients [53]. The recruitment of MDSC is significantly influenced by CXCR2, with its major ligands for chemotaxis including CXCL5, CXCL2, CXCL1, and CXCL8 [54]. Oka et al. [55] found that CXCL17 reduces IMQ-induced psoriasis-like skin inflammation by drawing MDSCs, which then promote regulatory T cells via CCL5 and CCL4. We found a strong correlation between MDSCs and CCL19 for the first time. This finding requires further validation through basic and clinical research. The growing understanding of MDSCs in psoriasis pathogenesis, particularly their interaction with chemokines like CCL19, unveils promising new therapeutic possibilities.

GSEA analysis indicated that the group with high expression of the six hub genes exhibited significant enrichment in the proteasome and DNA replication pathways. The proteasome, an essential component of the ubiquitin-proteasome system (UPS), is crucial for protein degradation and impacts a variety of cellular functions, including cell differentiation, proliferation, migration, angiogenesis, transcription activation, and immune responses [56]. The UPS is also implicated in the regulation of mammalian DNA replication [57]. Karabowicz et al. [58] demonstrated that the selective proteasome inhibitor PS-519 prevents IκB degradation and inhibits NF-κB downstream signaling, thereby reducing T-cell activation both in vitro and in vivo. In addition, it has been observed that elevated levels of CCL3 can exacerbate psoriatic lesions by promoting Foxp3 degradation in regulatory T cells through conventional K48-linked ubiquitination [59]. These findings led us to hypothesize that the proteasome pathway may play a significant role in the pathogenesis of psoriasis, particularly concerning the six chemokine family members (CCR7, CCL2, CCL19, CXCL8, CXCL1, and CXCL2). Further research is needed to explore this hypothesis in greater detail. Overall, our study offers new directions for future investigation in this area.

Previous studies have highlighted the therapeutic potential of deferoxamine and ABX-IL8 in managing psoriasis [60, 61]. Additionally, SCH 47112, a derivative of staurosporine, has shown a potential to reduce inflammation and proliferation in psoriatic conditions [62]. Our study introduces the first evidence suggesting that deferoxamine and staurosporine might mediate anti-inflammatory effects by specifically targeting CXCL2. Although carlumab and bindarit, inhibitors focusing on CCL2, have been considered for systemic sclerosis [63], their effectiveness in psoriasis has yet to be assessed. Clinical trials have confirmed the safety and feasibility of carlumab in solid tumors [64], and bindarit has shown potential in reducing periodontal inflammation [65], indicating possible therapeutic benefits in psoriasis. Pharmacokinetic assessments of the anti-IL-8 monoclonal antibody, HUMAX-IL8, recorded significant reductions in serum IL-8 levels with good tolerability [66], suggesting a promising approach for psoriasis treatment. In summary, our study sheds light on potential psoriasis therapies and paves the way for the development of new and effective treatments for the disease.

In the TF-miRNA-hub gene network analysis, our study identified 52 TFs and 139 miRNAs as the master regulators of the six co-expressed genes associated with psoriasis. More than 250 miRNAs have been reported to be aberrantly expressed in the skin or blood of patients with psoriasis [67]. Although miR-210 upregulation in psoriasis has previously been noted [67], the link between miR-210 and CXCL8 was first discovered in our research. Mostafa et al. [68] reported that the upregulation of miR-203 in psoriatic tissue could reduce the proinflammatory response by directly targeting and reducing CXCL8 expression. Our findings indicate increased CXCL8 expression, suggesting that elevated miR-203 levels might positively influence psoriasis by moderating CXCL8’s abnormal expression, or it might represent a compensatory response to the abnormal expression of CXCL8. These results suggest that miR-203 could play a regulatory role in the pathogenesis of psoriasis via its interaction with CXCL8, though further research is needed to clarify the involved mechanisms. Additionally, miR-155 was found to indirectly affect CXCL8 production, thereby influencing psoriasis progression [69], which supports our findings. miR-335, known to target the IL-17 cytokine pathway-related immune response in psoriasis [70], was implicated in regulating all six hub genes in our study, highlighting its importance in further research. Our study highlighted CCR7, CCL2, CCL19, CXCL8, CXCL1, and CXCL2 as potential inflammatory biomarkers in psoriasis, illuminating their molecular mechanisms. Our findings advocate for a detailed exploration of these biomarkers to aid in developing new therapeutic targets for psoriasis.

A notable limitation of our study though is the exclusive dependence on public database information, which could introduce heterogeneity into our results. To strengthen our conclusions, studies with larger sample sizes and broader experimental scopes are needed. Moving forward, it is crucial to address these limitations by conducting further research into the roles of these genes in psoriasis. We are committed to continuing our monitoring and investigation of these biomarkers to enhance our understanding of their impacts on psoriasis treatment and potentially on other inflammatory conditions as well.

## Conclusion

The study highlights six chemokine genes (*CCR7, CCL2, CCL19, CXCL8, CXCL1,* and *CXCL2*) as potential biomarkers in psoriasis, which are significantly involved in immune and inflammatory responses. These findings offer new insights into the pathogenesis of psoriasis and suggest chemokine-associated pathways as promising therapeutic targets.

## Supplemental data

Supplementary data are available at the following link: https://www.bjbms.org/ojs/index.php/bjbms/article/view/10327/3218.

## Data Availability

The original contributions presented in the study are publicly available. This data can be found here: GSE13355, GSE14905, and GSE78097.
